# Isotope Geochemistry for Seafood Traceability and Authentication: The Northern Adriatic Manila Clams Case Study

**DOI:** 10.3390/foods11193054

**Published:** 2022-10-01

**Authors:** Valentina Brombin, Claudio Natali, Gianluca Frijia, Katharina Schmitt, Martina Casalini, Gianluca Bianchini

**Affiliations:** 1 Department of Physics and Earth Sciences, University of Ferrara, Via Giuseppe Saragat 1, 44122 Ferrara, Italy; 2 Department of Earth Sciences, University of Florence, Via La Pira 4, 50121 Florence, Italy; 3 Institute of Geosciences, University of Mainz, Johann-Joachim-Becher-Weg 21, 55128 Mainz, Germany

**Keywords:** manila clams, *Ruditapes philippinarum*, seafood, traceability, northern Adriatic lagoons, isotopes

## Abstract

In Italy, the production of manila clams (*Ruditapes philippinarum*, Adams and Reeve, 1850) is mainly localized in northern Adriatic lagoons in the Po River delta, where shellfish farming provides important socio-economic revenue. However, in our globalized world, the seafood market is threated by fraudulent activities, in which agri-food products whose provenance is not certified are sold, posing a risk to consumer health. Multi-isotope ratio analysis is commonly used to trace the provenance of goods produced in different countries with different climatic and environmental conditions. Here, we investigated the reliability of this approach in terms of tracing the exact provenance of manila clams harvested in three Adriatic northern lagoons that are close to each other. We also verified the origin of samples bought at a local supermarket with a certificate of provenance. We carried out elemental analyses of carbon (C), nitrogen (N), and sulfur (S) and the respective isotopic ratios (^13^C/^12^C; ^15^N/^14^N; ^34^S/^32^S) on manila clam tissues, plus isotopic analyses of carbon (^13^C/^12^C), oxygen (^18^O/^16^O), and strontium (^87^Sr/^86^Sr) on manila clam shells. Each isotopic parameter can be used to identify the marine and continental contributions of water and/or nutrient supplies occurring in the lagoons. Therefore, the combination of isotopic parameters in a linear discriminant analysis (LDA) allowed for the identification of the lagoons in which the manila clams were produced.

## 1. Introduction

Food authenticity has become a major concern for consumers who want to be conscious about the origin of the products they eat. Food market globalization and the increasing ease of transport of food commodities between countries and continents has facilitated fraudulent activities in this area [1]. This misconduct poses a risk to consumer health and ethics, plus it affects the interests of retailers and food producers. In this context, it is important for both consumers and producers to have rapid and cost-effective analytical approaches to minimize the mislabeling of agri-food products. This is particularly urgent for the seafood market, which is commonly affected by food fraud driven by increasing market demand, resource limitations, value, and the complex supply chains [2]. In addition, seafood quality is influenced by the environmental conditions of the source habitat, which can be potentially affected by the input of organic pollutants, toxins, parasites, and heavy metals transferred into the trophic chain [2,3]. In this context, it is of paramount importance to set up an analytical approach that allows the geographical area of provenance of the products to be assessed. For product authentication, the most promising approach involves the use of the isotopic fingerprint of stable (C, N, O, H, S) and/or radiogenic (Sr) isotopes as tracers of the geographical provenance of agri-foods, e.g., olive oil, meat, honey, wine, and vanilla [4,5,6,7,8,9,10]. 

Recently, Won et al. [11] traced the country of origin of manila clams (*Ruditapes philippinarum*, Adams and Reeve, 1850), one of the most important seafood products farmed worldwide [12], on the basis of stable (H, C, N, O, S) and radiogenic (Sr) isotopic fingerprints. In a preliminary work, Bianchini et al. [13] demonstrated that the use of the elemental and isotopic compositions of C and N in the manila clams’ tissues and shells is a promising approach for the identification of the provenance of seafoods living in neighboring lagoons. However, temporal (from seasonal to multi-decadal) climatic variations and anthropogenic impacts can modify the hydrological environment of bivalves and their trophic resources, and in turn, their isotopic composition [14]. This is particularly true for C and N isotopes in tissues, as they tightly reflect the isotopic fingerprint of their food sources (particulate organic matter, microphytobenthos, and sediment organic matter). However, Won et al. [11] showed that they can be useful when combined with other main constituents, such as S in tissues and C and O of the shells, which are mainly related to environmental features (e.g., the temperature and composition of the water). This approach was only tested to assess the provenance of manila clams at the regional (country) scale, which is characterized by significantly different climatic and environmental conditions, but it was not tested in a small-scale area, where different farming habitats (e.g., lagoons) are in close proximity. In this work we used the sample suite of Bianchini et al. [13], who investigated Italian manila clams from northern Adriatic lagoons, to test whether a combined geochemical approach is also useful to trace manila clams that are from neighboring sites. Here, we report the complete sample dataset preliminarily studied by Bianchini et al. [15], who reported a summary (i.e., averaged data) of C and N elemental and isotope compositions of manila clam tissues measured using the elemental analysis-isotope ratio mass spectrometry (EA-IRMS) technique. In addition, we explored the application of new tracers and analytical methods, which could be useful for traceability studies, such as: (i) the S elemental and isotopic ratio (^34^S/^32^S) of clam tissues using the EA-IRMS technique; (ii) the C and O isotopic ratios (^13^C/^12^C and ^18^O/^16^O) of the aragonitic shells after the dissolution-equilibration of the samples using an IRMS; and (iii) the Sr isotopic ratios (^87^Sr/^86^Sr) of shells using the thermal ionization mass spectrometry (TIMS).

## 2. Materials and Methods

### 2.1. Study Area

**Figure 1:**
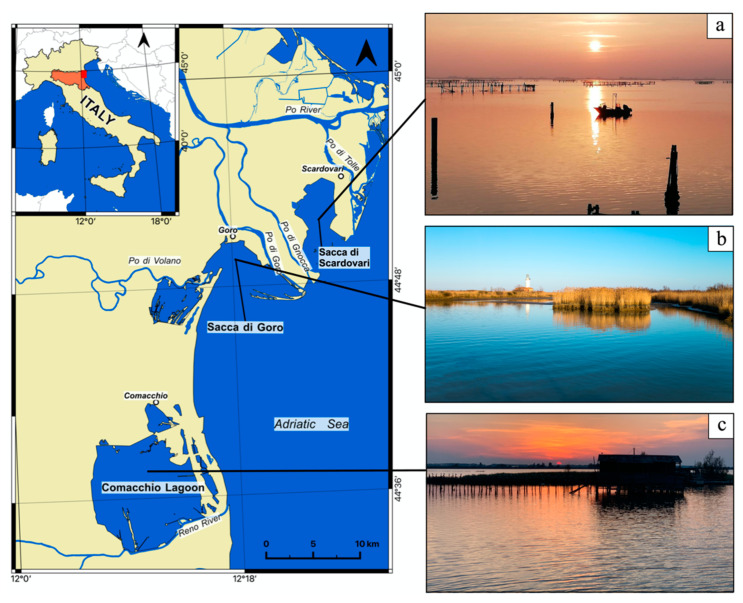
Geographic setting and locations of the investigated northern Adriatic lagoons within the Po River delta area in Italy. Photos of (**a**) Sacca di Goro; (**b**) Sacca di Scardovari; and (**c**) the Comacchio Lagoon.

The manila clams in this study were from three different coastal lagoons: Sacca di Goro (44.82° N, 12.31° E), Sacca di Scardovari (44.86° N, 12.42° E), and the Comacchio Lagoon (44.61° N, 12.17° E), which are located on the Italian northern Adriatic coast within the delta of the Po River, the main Italian fluvial system [14,15,16] (Figure 1). This area is characterized by a temperate climate with a mean annual precipitation of 666 mm, concentrated during the autumn, and by a mean annual air temperature of 24.5 °C. July and August are the warmest months and January is the coldest. Each lagoon receives nutrient-rich freshwater from different branches of the Po River. Both Sacca di Scardovari (Figure 1a) and Sacca di Goro (Figure 1b) are embayments connected to the Adriatic Sea through wide mouths, which facilitate the exchange of fresh and marine waters, an ideal feature for aquaculture. The Sacca di Scardovari receives a limited contribution of freshwater from small agricultural canals, whereas Sacca di Goro receives nutrient-rich freshwater from two branches of the Po River: Po di Goro and Po di Volano. The Comacchio Lagoon (Figure 1c) is a large, non-tidal lagoon that is not directly open to the sea. It is characterized by poor water oxygenation and by the accumulation of organic-matter-rich sediments. A more detailed description of the lagoons is reported in the work of Bianchini et al. [13].

### 2.2. Sampling

The manila clams in this study were collected in the northern Adriatic lagoons, where Italian clam production is concentrated. Ten samples were collected in Sacca di Goro and four in Sacca di Scardovari in May 2015 and May 2016, respectively. Further four samples from Sacca di Goro were obtained from a local fisherman in June 2018 and contemporarily four samples were bought from a local supermarket, exhibiting certified provenance for comparison. Before being put on sale, manila clams are generally purified through depuration processes in tanks of clean seawater to boost the filtering activity of the clams and promote the expulsion of their intestinal contents, including possible contaminants. Three samples were collected from the Comacchio Lagoon in September 2017. The size of all the sampled shells was between 2 and 3 cm in length and the dry weight of the tissues was between 1 and 2 g.

### 2.3. Preparation of the Samples and Measurement Methods

After drying the samples at 60°C in an oven following the procedure of Yokoyama and Ishihi [17], the shells were separated from the clam tissues, washed, and brushed with hydrogen peroxide. This sample treatment was necessary to eliminate superficial organic residue that can affect the C and O isotopic composition of the shell [18]. Both tissues and shells were subsequently powdered in an agate mill and analyzed for elemental contents and isotopic ratios. The C, N, and S elemental and isotopic analyses of tissues were performed with specific elemental analyzers operating in combustion mode at controlled temperatures coupled with continuous flow isotope ratio mass spectrometers (EA-IRMS). The bulk C and O isotopic analyses of carbonate shells were performed with a continuous flow isotope ratio mass spectrometer (IRMS; Elementar©, Cheadle, UK) coupled with a headspace analyzer (HA; Elementar©, Langenselbold, Germany) for the determination of the CO_2_ gas released after the reaction of the carbonate matrix with orthophosphoric (H_3_PO_4_) acid. In addition, shells were analyzed for their Sr isotopic composition by using a thermal ionization mass spectrometer (TIMS; Thermo Fisher©; Bremen, Germany) following chromatographic separation. The analytical details are reported in the following sections.

#### 2.3.1. C, N, and S Elemental and Isotopic Analyses of Manila Clam Tissues

For the analyses of C, N, and S contents and the relative isotope ratios (^13^C/^12^C, ^15^N/^14^N, ^34^S/^32^S) of clam tissues, 5–10 mg of homogenous powder was analyzed. The analyses of C and N were carried out in the Laboratory of Isotopic Geochemistry of Soils and Food of the Department of Physics and Earth Science of University of Ferrara using an elemental analyzer (EA) VarioMicro Cube (Elementar©, Hanau, Germany) operating in combustion mode and coupled with the isotope ratio mass spectrometer (IRMS) Isoprime100 (Isoprime©, Manchester, UK). The detailed analytical methods are reported in the work of Bianchini et al [13].

Additional analyses were performed for the clam tissues to measure the S content and isotopic ratio. These analyses were carried out in the Laboratory of Isotopic Geochemistry for the Environment and Georesources of the Department of Physics and Earth Science of University of Ferrara using an EA Vario PYRO Cube (Elementar©, Langenselbold, Germany) coupled with the IRMS PrecisION (Elementar©, Cheadle, UK). For each sample, powder was weighed in tin capsules, wrapped, and finally loaded in the EA autosampler to be analyzed. Samples were burnt via flash combustion with O_2_ at 1150 °C and the released S gaseous species were transferred in a reduction column operating at 850 °C to remove the excess of oxygen. After the elemental analysis, the SO_2_ gas was deposited in in the IRMS for the isotopic analysis.

Calibration of the instrument was performed using several standards: the limestone JLs-1 [19], the Carrara Marble [20], the Jacupiranga carbonatite [21], the peach leaves NIST SRM1547 [22], the caffeine IAEA-600, the Tibetan human hair powder USGS42 [23], the Barium Sulphate IAEA-SO-5 [24].

The ^13^C/^12^C, ^15^N/^14^N and ^34^S/^32^S isotopic ratios (R) were expressed with the δ notation (in ‰ units):

(1) $$\delta \;=\;\left(\frac{R_{\mathrm{sam}}}{R_{\mathrm{std}}}-1\right)\times 1000$$

where R_sam_ is the isotopic ratio of the sample and R_std_ is the isotopic ratio of the international isotope standards Vienna-Pee Dee Belemnite (V-PDB), Air N_2_, and Vienna-Canyon Diablo troilite (V-CDT) for C, N, and S, respectively. Analytical uncertainties (1σ) for the isotope analyses were in the order of ± 0.1‰ for δ^13^C and ± 0.3‰ for δ^15^N and δ^34^S, as indicated by repeated analyses of samples and standards.

#### 2.3.2. C and O Isotopic Analyses of Manila Clam Shells

The isotopic analyses of C and O for manila clam shells were performed at the Laboratory of Paleoclimatology and Isotopic Stratigraphy of the Department of Physics and Earth Sciences of the University of Ferrara, using an isoFLOW headspace analyzer (HA, Elementar©, Langenselbold, Germany) operating in continuous flow with a PrecisION IRMS (Elementar©, Cheadle, UK). For each analysis, approximately 150 µg of homogenous powder of the sample was weighed and put into vials. In each vial, the powder was reacted with hot viscous water-free orthophosphoric (H_3_PO_4_) acid for 3 h at 50 °C, triggering the release of CO_2_ from the carbonate shell material. The generated CO_2_ gas was transferred to the IRMS for the simultaneous measurement of the C and O isotopic ratios. For this analysis, a single-point calibration with the in-house MAQ-1 standard was used, whereas IAEA 603 was used as the control standard. Analytical uncertainties (1σ) for the isotope analyses were in the order of ±0.1 ‰ for δ^13^C and δ^18^O. The ^13^C/^12^C and ^18^O/^16^O isotopic ratios were expressed with the δ notation (in ‰ units) relative to V-PDB.

#### 2.3.3. Sr Isotopic Analyses of Manila Clam Shells

Strontium isotope analyses on manila clam tissues were carried out at the Department of Earth Sciences of the University of Firenze. For each group, a subset of samples was selected: three for Sacca di Goro (2015), Sacca di Scardovari, and the Comacchio Lagoon, two for Sacca di Goro (2018), and one for the supermarket.

Approximately 20 mg of each sample was totally digested in PFA Savillex beakers with a mixture of ultrapure HCl and HNO_3_ for ca. 24 h on a hot plate. The Sr purification was ensured through chromatographic separation using Eichrom Sr-Spec resin (150 μL). The sample was loaded as 3N HNO_3_ solution on the resin bed and washed several times with 3N HNO_3_, allowing the elution of most of the matrix elements. Sr was finally recovered in MilliQ, dried down, and refluxed at high temperature (150 °C) twice with concentrated HNO_3_ and H_2_O_2_ to ensure the removal of any organic residue from the resin. The complete procedure for chromatographic separation is described in detail in the work of Avanzinelli et al. [25]. The isotope measurements were performed loading approximately 250 ng of sample onto single Re-filament as nitrate form with TaCl_5_ and H_3_PO_4_ as activator and to keep the signal stable during the measurements. The Sr isotope ratios were measured in multi-dynamic mode, applying the triple jump procedure described in detail by Avanzinelli et al. [25]. Each reported isotope ratio is the result of 120 cycles (with each cycle representing the average of three measurements performed during the triple-jump procedure), taken in 6 blocks, each consisting of 20 cycles with 8 s integration time. The instrumental mass bias was corrected off-line using the ^88^Sr/^86^Sr ratio measured on the main configuration (jump 2). The measured and the natural ^88^Sr/^86^Sr (^88^Sr/^86^Sr_N_ = 8.375209) were used both to calculate the mass discrimination factor (ε) and to subsequently apply the correction through the exponential fractionation law. The precision of the method was guaranteed by the reiterate analyses of several certified materials run in the same lab (e.g., AGV-1, BHVO-1), which were all in agreement with the literature [26]. The ^87^Sr/^86^Sr average value for the NIST SRM987 international reference standard over the period of analysis was within the error of the long-term reproducibility, i.e., 0.710250 ± 0.000023 (2σ, *n* = 126), and in agreement with the values reported in Thirlwall [27].

### 2.4. Statistical Analysis

The statistical analysis was conducted using the R package (version 4.0.2; R Core Team; R Foundation for Statistical Computing; Vienna, Austria; [28]). The analysis of variance (ANOVA test) was applied to every variable to determine the statistical differences between clams from distinct lagoons. Pairwise comparisons were performed using Tukey’s HSD (honestly significantly different) test. Linear discriminant analysis (LDA) was applied to maximize the separation of known categories and to trace the exact lagoon of provenance of the manila clams. The linear discriminant function was validated via the leave-one-out cross-validation (LOOCV) method.

## 3. Results

**Figure 2:**
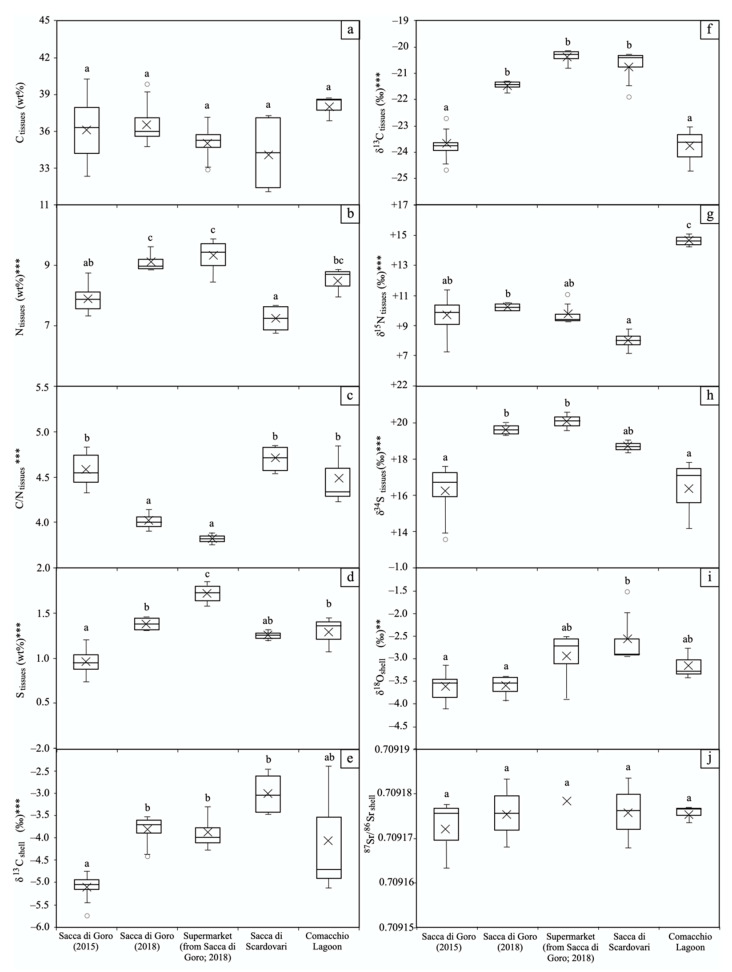
Box plot of elemental and isotopic compositions of carbon (C), nitrogen (N), sulfur (S), oxygen (O), and strontium isotope composition (^87^Sr/^86^Sr) in the tissues (**a**–**d**; **f**–**h**) and shells (**e**; **i**–**j**) of manila clam samples collected on-site from northern Adriatic lagoons and bought at the local supermarket. In each box plot, the black line and the black cross represent the median and the mean, respectively. Above the box plots, the letters represent the results of the Tukey’s HSD post hoc test: different letters denote significant differences among groups. The one-way ANOVA results are also reported (** *p* < 0.001; *** *p* < 0.0001); where the asterisks are missing the groups are not statistically different.

The elemental contents of C, N, S, and the relative isotopic ratios of the manila clam tissues, together with the C, O, and Sr isotopic ratios of shells, are reported in Table 1 and Figure 2.

For the manila clam tissues the C elemental content did not exhibit significant variation (Sacca di Goro 2015: 32.2–40.3 wt%; Sacca di Goro 2018: 34.7–39.8 wt%; Sacca di Scardovari 2016: 32.8–37.1 wt%; Comacchio Lagoon 2017: 36.8–38.7 wt%; Table 1) and, according to the one-way ANOVA test, it was not statistically affected (*p*-value > 1) by the geographical area or the year of sampling (Figure 2a). On the other hand, the N content exhibited a large variability among the manila clam groups (Table 1; Figure 2b), with samples from Sacca di Goro collected on-site and bought at the local supermarket in the same year (2018) exhibiting similar N values (Sacca di Goro 2018: 8.9–9.6 wt%; supermarket: 8.5–9.9 wt%; Table 1). The N elemental contents of clam tissues were significantly affected (*p* < 0.001) by the lagoon of provenance and the year of sampling. For all manila clams, the C/N was higher than 4 (4.2–4.9, Table 1; Figure 2c), which is indicative of similar lipid contents [29,30]. However, those from Sacca di Goro and the ones bought at the supermarket in the same year (2018) display lower C/N values (3.9–4.1 and 3.8–3.9, respectively, Table 1), indicating minor lipid content.

In comparison to N, the range of S elemental content in clam tissues among the sample groups was more limited, with the exception of manila clams from Sacca di Goro collected in 2015 and those bought at the supermarket in 2018, which exhibited the lowest (0.7–1.2 wt%; Table 1; Figure 2d) and the highest (1.6–1.9 wt%; Table 1; Figure 2d) values, respectively, of the entire sample suite. As for N, the S elemental contents of clam tissues were also significantly affected (*p* < 0.001) by the lagoon of provenance and the year of sampling (Figure 2d). 

All the isotopic ratios of C, N, and S in manila clam tissues were significantly affected (*p* < 0.001) by the lagoon of provenance and/or the year of sampling according to the ANOVA test (Figure 2f–h), indicating that at least one group of manila clams was statistically different from the others. The Tukey post hoc test better explored the similarities and differences among the manila clam groups (Figure 2f–h). In detail, the δ^13^C values of manila clams from Sacca di Goro (2018), Sacca di Scardovari, and those bought at the supermarket were similar (−21.7 to −21.3‰, −21.9 to −20.4‰, −20.8 to −20.2‰, respectively; Table 1; Figure 2f) but less negative than those of Sacca di Goro (2015) and the Comacchio Lagoon (−24.7 to −22.7‰, −24.7 to −23.0‰, respectively; Table 1; Figure 2f). The δ^15^N values of manila clams from Sacca di Goro (2015), Sacca di Goro (2018), the supermarket, and Sacca di Scardovari overlapped with each other (+7.3 to +11.4‰; +10.1 to +10.5‰; +9.3 to +11.1‰; +7.2 to +8.8‰, respectively; Table 1; Figure 2g), but the δ^15^N signature of manila clams from the Comacchio Lagoon was much more positive (+14.2 to +15.2‰; Table 1; Figure 2g). The δ^34^S values of manila clams from Sacca di Goro (2018) and from the supermarket collected in the same lagoon and in the same year were similar (+19.2 to +19.9‰, +19.5 to +20.5‰, respectively), but they differed from the others (Sacca di Goro, 2015: +13.5 to +17.5‰; Sacca di Scardovari: +18.3 to +19.0‰; Comacchio Lagoon: +14.2 to +17.8‰; Table 1; Figure 2h).

For the aragonite shells of the manila clams, ANOVA test and Tukey’s post hoc test were applied for the isotopic ratios of C, O, and Sr. Among the isotopic parameters, the δ^13^C was the most statistically affected (*p* < 0.001) by the lagoon of provenance and the year of sampling (Sacca di Goro, 2015: −5.8 to −4.8‰; Sacca di Goro, 2018: −4.4 to −3.5‰; supermarket: −4.3 to −3.3‰; Sacca di Scardovari: −3.5 to −2.5‰; Comacchio Lagoon: −5.1 to −2.4‰; Table 1; Figure 2e). In comparison to C, the O isotopic ratios were less affected (*p* < 0.01) by the lagoon and/or the year of the sampling, as the ranges of δ^18^O of the manila clam groups overlapped with each other (Sacca di Goro, 2015: −4.1 to −3.2‰; Sacca di Goro, 2018: −3.9 to −3.4‰; supermarket: −3.9 to −2.5‰; Comacchio Lagoon: −3.4 to −2.8‰; Table 1; Figure 2i), with the exception of samples from Sacca di Scardovari which exhibited less negative values (−3.0 to −1.5‰; Table 1; Figure 2i). Finally, despite the limited number of analyses, the Sr isotopic ratio was not significantly affected (*p* > 1) by the provenance and/or the year of collection of the bivalves, as shown by its limited variation among the whole sample dataset (Sacca di Goro, 2015: 0.709163–0.709178; Sacca di Goro, 2018: 0.709168–0.709183; supermarket: 0.709179; Sacca di Scardovari: 0.709168–0.709184; Comacchio Lagoon: 0.709173–0.709177; Table 1; Figure 2j).

## 4. Discussion

Tracing the geographic origin of organic substances involves the measurements of isotopic signatures, typically of light common elements such as ^13^C/^12^C, ^15^N/^14^N, ^18^O/^16^O, and ^34^S/^32^S, as well as radiogenic isotopic ratio including ^87^Sr/^86^Sr [1]. As the enrichment or depletion of these isotopic ratios in any organism is affected by its metabolic activity and the features of the surrounding environment (e.g., climate, geological features, and anthropogenic impacts), the recorded isotopic signatures are considered as valuable tools for the traceability of many food products. Thus, seafood of the same species produced in different geographical regions with diverse environmental conditions exhibits uniquely assignable isotopic signatures [11,31,32,33]. We verified the applicability of this method at a small scale by investigating the variation in the isotopic signature among manila clams collected in neighboring lagoons with similar climatic and environmental conditions. In the following sections, we discuss the potential factors that affect each isotopic parameter, and we evaluate the contribution of each element as a potential provenance tracer of manila clams.

### 4.1. C and N Isotopic Ratios of Manila Clam Tissues

**Figure 3:**
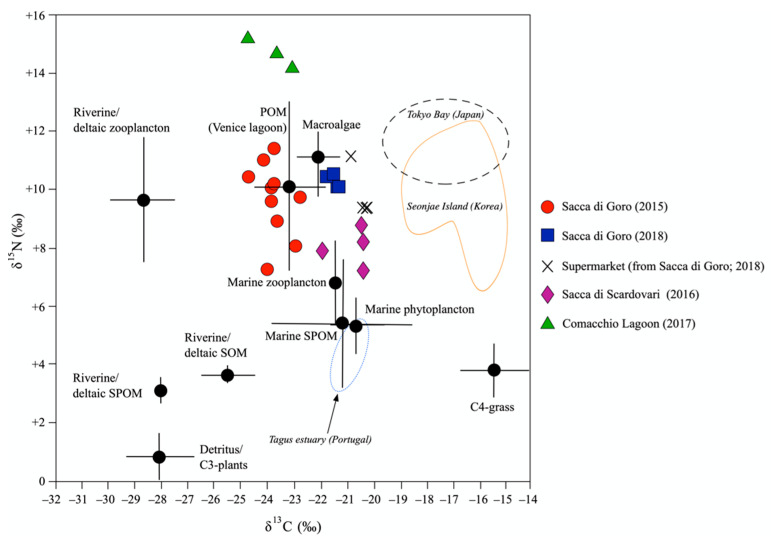
Comparison of carbon (C) and nitrogen (N) isotopic compositions of manila clam tissues from northern Adriatic lagoons with those from (i) fisheries worldwide (Tagus estuary, Portugal [30]; Tokyo Bay, Japan [44]; Seonjae Island, Korea [45]) and with (ii) potential organic sources collected in the Po delta area [38] and in the Venice lagoon [39]. The analytical uncertainties are negligible at the graphical scale.

Most of the studies addressing the topic of the traceability of animals are mainly focused on the C and N isotopes in tissues, as they record the isotopic composition of the consumed food, which is, in turn, linked to the features of the environment [34,35]. Analogously, C and N isotopes were successfully used to distinguish manila clams living in different countries [13] (Figure 3), as they are suspension-feeding bivalves that primarily feed on particulate organic matter (POM), and subordinately on phytoplankton and benthic microalgae [29,36,37]. In intertidal areas, POM is derived from heterogeneous sources including both autochthonous and allochthonous organic matter, sediment, seagrass, and seaweeds [37]. In addition, in delta areas, the inputs of organic matter are rivers, which transport terrestrial material (i.e., plant detritus, soils) and anthropogenic inputs (i.e., wastewater) [29,38]. The manila clam samples in this study were collected in a lagoon setting of a large delta system, therefore they potentially received POM of both terrestrial and marine provenance, which are characterized by distinct isotopic signatures. The relative contribution of terrestrial and marine nutrients depends on several factors, one of them being the seasonal fluctuations of the river flow. During the dry season, the nutrients of marine origin are predominant in the lagoons because of the low river discharge. On the contrary, during the wet season the contribution of nutrients of terrestrial origin is strong because of the high river flow [29,38]. The manila clams in this study were sampled during the end of spring and summer, therefore the marine contribution to POM was expected to be predominant. Accordingly, in Figure 3, the C and N isotopic ratios of manila clams from Sacca di Goro, the supermarket, and Sacca di Scardovari were similar to those of the POM from the Venice lagoon (another northern Adriatic lagoon in the Po delta area) sampled in May, when marine contributions prevail [39]. Taking into account that for *Ruditapes philippinarum* the trophic enrichment factors are +0.6‰ and +3.4‰ for ^13^C and ^15^N, respectively [40], in Figure 3, the manila clam samples were closer to the marine inputs (i.e., suspended POM and marine phytoplankton) than to the terrestrial contributions collected in the delta areas of the Po River and investigated by Bongiorni et al. [38]. Only the C and N isotopic compositions of manila clams from the Comacchio Lagoon were well distinguishable from those of the other northern Adriatic lagoons, indicating a different source of nutrient discharge. Another factor that affects the relative contribution of nutrients of marine and/or terrestrial origin is the geomorphology of a lagoon, in particular the degree of confinement of the lagoons from the sea and the connection with the river network. The Comacchio Lagoon is a confined aquatic ecosystem with a longer water retention time (mean: 247 days; [41]) compared to that of the nearby open lagoons Sacca di Goro and Sacca di Scardovari (mean: 3 days; [41]). Consequently, the POM of the Comacchio Lagoon is mainly characterized as being of terrestrial provenance, whereas the POM of Sacca di Goro and Sacca di Scardovari includes both marine and terrestrial contributions, as these lagoons supply waters and nutrients by both the Adriatic Sea and the Po River. In addition, the Comacchio Lagoon manila clams exhibited the highest δ^15^N isotopic values, which are indicative of eutrophication events caused by anthropogenic impacts [42]. In fact, high δ^15^N values are typical of animal waste, i.e., the natural nitrogenous fertilizers mainly used in farms [43]. All the northern Adriatic lagoons located nearby those investigated in this study are affected by the agricultural runoff dragged by the Po River, since it drains the Po plain that hosts 35% of Italian agricultural production [41]. However, as the Comacchio Lagoon is poorly connected to the sea, the exchange with seawater is limited, causing an accumulation of terrestrial nutrients that may trigger eutrophication events [42]. Therefore, aside from traceability studies, the continuous monitoring of C and N isotopic composition of manila clams could also be applied for the reconstruction of the nutrient provenance and the impact of nutrients on the environment, which are useful to indirectly evaluate the water quality and aquatic ecosystems.

It is evident that C and N isotopic ratios are suitable tracers of the potential food source of manila clams, and, in turn, of the geographic origin of the manila clams at the regional (country)-scale. However, these two parameters do not guarantee a clear discrimination of the origin of the organism at the local scale, i.e., when niches of provenance are just a few kilometers apart (see [13]), because lagoons mainly share the same input of nutrients. Therefore, for such “level of detail”, alternative/additional parameters should be investigated.

### 4.2. S Isotopic Ratios of Manila Clam Tissues

**Figure 4:**
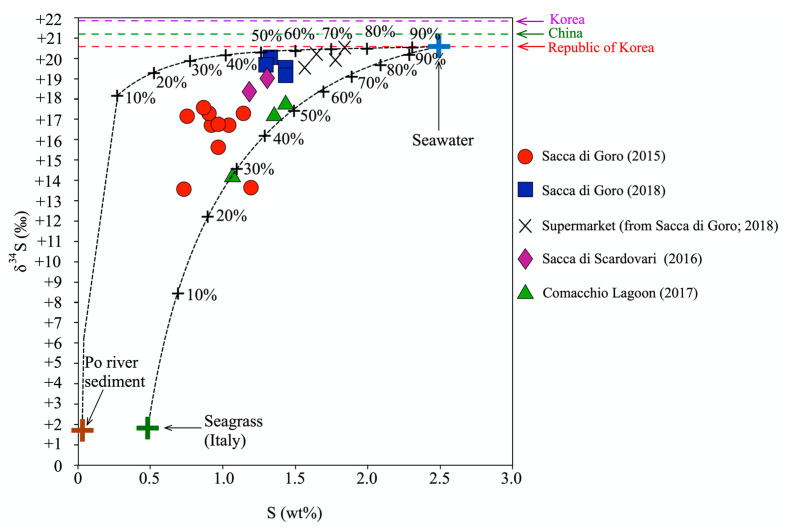
Sulfur (S) content vs. δ^34^S of tissues of manila clams collected on-site from northern Adriatic lagoons and bought at a local supermarket. The elemental and isotopic values of S for seawater are from Adriatic marine plankton of Giani et al. [54] and Borrell et al. [55], respectively. The values for Po River sediments are from Salani et al. [56], and for seagrass, they are from Holmer and Nasler-Sheetal [57]. Tick marks on the seawater–riverine mixing lines indicate the contribution of seawater. The average δ^34^S values from China, Democratic People’s Republic of Korea (DPR), and Korea from the study of Won et al. [11] are also represented for a comparison. The analytical uncertainties are negligible at the graphical scale.

The S isotopic signature is not commonly used in traceability studies, despite the sulfur in animal tissue being related to the diet, with little (≤1‰) to no trophic fractionation [46,47,48]. In the literature, the S isotopic signature of aquatic animal tissues was explored as an alternative parameter for the determination of potential food sources and of the habitat type of aquatic organisms, i.e., marine vs. freshwater [49,50,51,52]. Generally, the S isotopic signature of organisms’ tissues is easy recognizable, as the δ^34^S values differ between marine and freshwater environments [51]. The seawater δ^34^S values consistently tend to be approximately +20‰ with S in form of sulphates enriched in ^34^S [49], whereas the freshwater δ^34^S values vary from −5 to +10‰ in relation to the watershed geology, anthropogenic inputs, and atmospheric deposition [53]. In this context, the variation in the S isotopic ratio in the manila clam tissues in our study (δ^34^S from +13.5 to +20.5‰; Table 1) is indicative of the relative marine and freshwater contributions in the lagoons, which represent a peculiar feature of these environments. In fact, their unique fingerprint is distinguishable from other manila clams from the coasts of China, Democratic People’s Republic of Korea, and Republic of Korea, as reported in the literature (+20.4 to +21.9‰; [11]), which are grown in a marine environment. This implies that S is a valid discriminant element for an aquatic organism’s traceability at least on the macro-scale. Interestingly, the elemental and isotopic S variations also allow the differentiation of manila clam samples from the northern Adriatic lagoons on the basis of the predominant origin (marine and continental) of nutrients that characterized the investigated water bodies in the different sampling periods. Figure 4 shows how distribution of clam tissues is controlled by the elemental and isotopic S composition of: (i) seawater, dominated by the Adriatic phytoplankton [54,55]; (ii) Po River sediments [56]; and (iii) seagrasses, which incorporate both sedimentary and marine sulfur [57] and represent a food source for the manila clams [37]. The variable contribution of the algal component to the organic matter content of bottom sediments from Sacca di Goro and Sacca di Scardovari was already reported by Natali and Bianchini [58]. In Figure 4, the elemental and isotopic S compositions of manila clams are plotted in the field defined by seawater [54,55], Po River sediments [56] and seagrass [57] end-members. The trend depicted by the samples mimics those of the two mixing lines, testifying to a correlation between the S composition of tissues and the different contributions of S sources in the lagoons. In particular, the manila clams from Sacca di Goro (2018), from the supermarket, and those from Sacca di Scardovari were characterized by the highest S contents coupled with ^34^S-enriched isotopic values (from +18.3 to +20.5‰, Table 1), close to the typical signature of marine organisms (around +20‰). Our model shows that these samples are related to a strong contribution of marine waters, reflecting their geomorphological features, including their connections with the Adriatic Sea. On the other hand, the δ^34^S values of manila clam tissues from the Comacchio Lagoon were distinctly lower and characterized by a higher variability (+14.2‰ to +17.8‰; Table 1). According to our model, these samples were less affected by the marine input, confirming the limited exchange between the Comacchio Lagoon and the Adriatic Sea. Moreover, in the diagram of Figure 4, manila clams from the Comacchio Lagoon are plotted on the mixing line between seawater and Italian seagrass, confirming the largest contribution of the latter as the S source of all the investigated sites, probably in response to the low hydrodynamics of the Comacchio Lagoon waters. Interestingly, the tissues of manila clams from Sacca di Goro sampled in 2015 exhibited the lowest S content (0.74–1.20 wt%) coupled with the most depleted S signature (+13.5‰ to +17.5‰). This could be related to the prevalent riverine contribution to the Sacca di Goro lagoon during the high Po River water flow in spring 2015.

In summary, the δ^34^S isotopic ratio of manila clam tissues can potentially be used to discriminate the provenance of the samples at both the regional and local scale. In addition, it is effective in distinguishing manila clams originating from open or closed lagoons and to record the environmental modifications induced by the occurrence of dramatic climatic events.

### 4.3. C and O Isotopic Ratios of Manila Clam Shells

**Figure 5:**
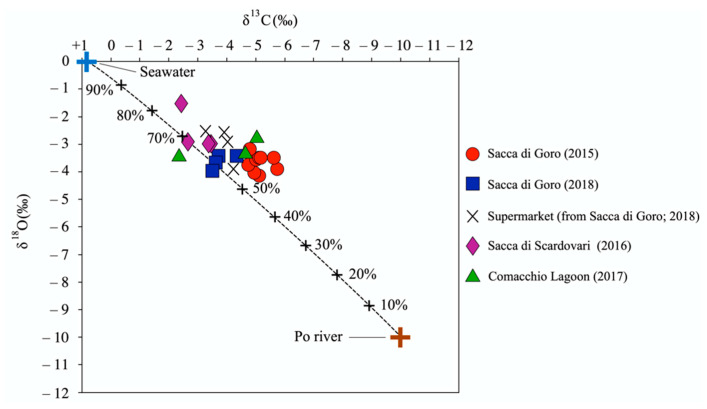
The δ^13^C and δ^18^O isotopic ratios of shells of manila clams collected on-site from northern Adriatic lagoons and bought at a local supermarket. The isotopic ratios of C of dissolved inorganic carbon (DIC) for seawater and the Po River are from Hoffman and Lamothe [64] and Marchina et al. [15], respectively, whereas the isotopic O of seawater is the typical value for Standard Mean Ocean Water (SMOW), and that of the Po River is from Marchina et al. [15]. Tick marks on the seawater–riverine mixing line indicate the contribution of seawater. The analytical uncertainties are negligible at the graphical scale.

Stable carbon and oxygen isotopes of carbonate shells are generally used for paleoenvironmental reconstructions (e.g., temperature, salinity, and productivity) but they are not usually employed for traceability studies. According to the statistical tests (Figure 2i), the O isotopic ratio of the shells is only slightly efficient in discriminating the provenance of the manila clams at the local scale. In fact, despite some differences that were present among the absolute δ^18^O values of the manila clam groups, their relative ranges mostly overlapped (Table 1; Figure 2). The O isotopic ratio of shells is a function of water salinity and temperature at the time of the shell formation [59,60,61,62]. These features hardly differ among northern Adriatic nearby lagoons. On the contrary, the δ^13^C values of shells can potentially represent a tracer of provenance of the clam, as feeding experiments demonstrated that the shell C isotope composition of *Ruditapes philippinarum* was influenced by both the HCO_3_^−^ content in seawater and the diet of the bivalve, the latter causing a shift toward negative values typical of the organic matter [63]. Coherently, the investigated manila clam shells showed a ^13^C-depleted composition with respect to the values defined by the mixing line connecting the marine and riverine dissolved inorganic carbon (DIC), demonstrating that they incorporated isotopically light (^12^C) metabolic carbon (Figure 5). In Figure 5, samples clustering with respect to the lagoon of provenance can be observed, reflecting different proportions of marine and freshwater inputs. In particular, the C and O isotopic signatures of manila clams from Sacca di Scardovari are indicative of a strong marine contribution (up to 80%; Figure 5), as this lagoon mainly receives water from the sea (Figure 1). On the contrary, the manila clams of Sacca di Goro (2015) and the Comacchio Lagoon were characterized by ^13^C- and ^18^O-depleted compositions, demonstrating the incorporation of a significant riverine contribution during their growth. The manila clams of Sacca di Goro (2018) instead showed a comparatively ^13^C-depleted composition, indicating that the lagoons received a minor input by the Po River in that year, especially in terms of suspended load. Interestingly, the samples bought at the supermarket had less negative δ^18^O values than the manila clams collected on-site in the lagoons, therefore the two groups only partially overlap in Figure 5. This could be due to the sold clams having a slightly different sampling location, e.g., toward the lagoon mouth facing the Adriatic Sea.

### 4.4. Sr Isotopic Ratios of Manila Clam Shells

**Figure 6:**
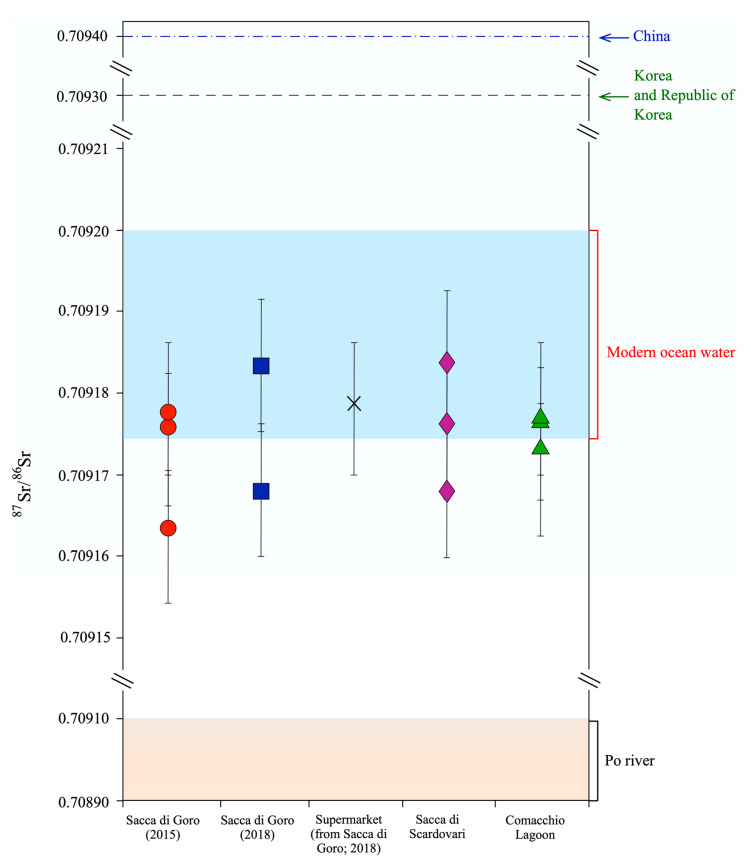
The ^87^Sr/^86^Sr isotopic ratios of shells of manila clams collected on-site from northern Adriatic lagoons and bought at a local supermarket. The isotopic ratios of manila clam tissues from China, Democratic People’s Republic of Korea, and Korea [11], modern ocean water range [68,69,75], and Po River range [66,67] are reported for comparison. The errors bars of the analyses are also represented.

The Sr isotope ratio is a geochemical tracer that is not affected by kinetic fractionations through biological processes, thus it reflects the relative contributions of Sr sources that are available in the habitat during the formation of these organisms [11]. The Sr isotopic composition of the studied manila clam shells was quite homogeneous (at least until the fifth decimal digit), with values ranging between 0.70916 and 0.70918. Relatively lower values (down to 0.7090) were recorded in shells from the Po proto-delta area during the Holocene, which correlated with the significant fluvial influence of coastal waters [65]. The Sr isotope composition of the Po River water after the closure section (located at Pontelagoscuro) mainly reflects the geological features of the basin, which are metamorphic/granitoid rocks (^87^Sr/^86^Sr > 0.7100) and carbonates (^87^Sr/^86^Sr < 0.7080) cropping out in the Po River catchment [66]. In fact, an intermediate value between those of the two geological end-members characterizes the present-day waters of the terminal part of the Po river (0.7089–0.7091; [66,67]). Although the investigated lagoons are characterized by different marine and continental contributions, as indicated by the S isotopic ratio of the manila clam tissues and the C and O stable isotopic ratios of the shells, the Sr isotopic composition of the investigated manila clams clustered around a median value of 0.709176 (Figure 6), which, considering the analytical uncertainty, roughly corresponds to the Sr isotopic composition of modern seawater [68,69]. This suggests that despite the variable influx of riverine Sr into the different lagoons, the Sr isotopic composition of waters, and in turn of the studied manila clam shells, mainly reflect the contribution from marine Sr. This may be justified considering the high Sr marine inventory (around 7 mg/L, [70]) and the low Sr concentration of the Po River (up to 300 ug/L; [66]). The Sr signature of clam shells from northern Adriatic lagoons was significantly lower than that recorded in analogous *Ruditapes philippinarum* tissues from China (average: 0.7094; [11]), Democratic People’s Republic of Korea, and Korea (average: 0.7093; [11]) (Figure 6). The Asiatic manila clams exhibited a comparatively more radiogenic Sr isotopic composition because of the significant impact of their riverine systems on the shellfish farming area, which is located in ponds in intertidal areas along the coast [71]. Continental waters significantly contribute to the coastal water budget in these environments and are characterized by very high ^87^Sr/^86^Sr values draining metamorphic and granitoid orogenic rocks (e.g., [72,73,74]). It is important to note that even if the Sr isotopic ratios were measured on different parts of the bivalve (i.e., tissues and shells), they are always comparable, as the Sr isotopes are not affected by biological fractionation. In summary, aquatic organisms growing in coastal areas record a Sr isotopic composition that is derived from the mixing of the continental weathering, which is, in turn, related to the regional geological features and the ocean water signals. Therefore, the Sr isotopic signature certainly represents a good parameter for the traceability of manila clams at the regional scale but is not suitable to trace the provenance at the local scale, such as in neighboring lagoons that share the same hydrogeological basin.

### 4.5. Multivariate Analyses for the Traceability of Manila Clams at the Local Scale

**Figure 7:**
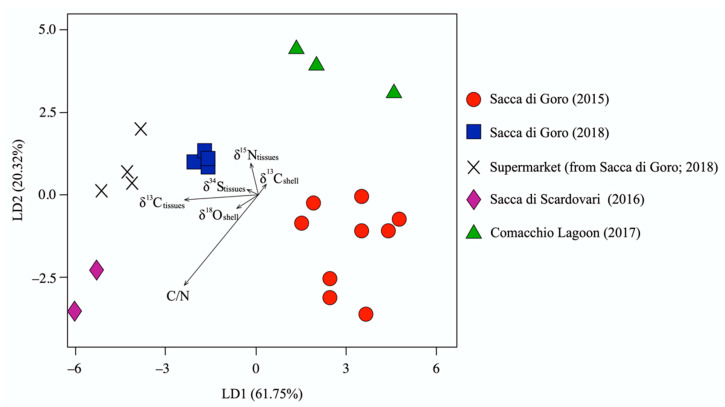
Linear discriminant analysis (LDA) of δ^13^C, δ^15^N, C/N, and δ^34^S C/N of tissues and δ^13^C and δ^18^O of shells of manila clams collected on-site from northern Adriatic lagoons and bought at a local supermarket.

In the previous sections, we explained that each isotopic parameter of manila clams variously reflects the environmental conditions and/or food sources of the lagoon of provenance, precluding the possibility of their use alone as exclusive provenance tracers at the local (small) scale. However, we explored the potential of their use in clam traceability when combining the information provided by each tracer using a multivariate statistical approach. In this framework, an LDA was performed to discriminate the lagoons of provenance of the manila clams. LDA is a statistical approach that maximizes the separation of known categories [76] and is one of the most popular models for food authentication [77]. To perform the LDA, we chose the C/N and the stable isotopic ratios as parameters, excluding the Sr isotope, as the differences in ^87^Sr/^86^Sr values among the manila clam groups is not relevant. The validation of the model was obtained by the LOOCV procedure. The resulting LDA explains 82% of the total variance, with linear discriminant 1 (LD1) accounting for 61.75% and linear discriminant 2 (LD2) for 20.32% (Figure 7). In the LDA plot (Figure 7), the manila clam samples are well clustered into five groups reflecting the lagoon of provenance and, in the case of Sacca di Goro, the year in which the clams were collected. Coherently, the clams bought at the supermarket and those collected in Sacca di Goro in 2018 were plotted in the same area of the diagram, with δ^13^C, δ^15^N, and C/N of the manila clam tissues being the most discriminant parameters among the two groups. Therefore, the C and N compositions of the different (marine and continental) food sources seem to play a crucial role in tracing manila clams at the local scale. However, the exclusive use of the C and N isotopic ratios of tissues does not allow one to trace the exact lagoons of provenance of the samples (Figure 3). The apparent small contribution provided by the δ^34^S values of tissues, and the δ^13^C and δ^18^O values of the shells, is therefore fundamental to separate the groups, as these parameters are related to the environmental settings, which are characteristic and unique for each lagoon. In order to take into account all of the different contributions provided by these variables for the traceability of manila clams, multivariate analysis certainly represents the best statistical tool, especially in terms of highlighting differences at the local scale. The LDA also shows that samples collected in Sacca di Goro in 2015 and 2018 are well clustered in two distinct groups, demonstrating that samples from the same location but collected in different years are characterized by significantly different C, N, O, and S isotopic signatures. This means that the variability of climate and environmental factors plays a crucial role in the manila clam isotopic compositions. Therefore, for the correct authentication of the seafood products, an exhaustive archive that reports the isotopic signatures of products from different locations should be created and annually updated.

## 5. Conclusions

Seafood traceability is critical to deterring illegal practices, verifying environmental and social responsibility claims, and supporting local and sustainable seafood producers. The multivariate analysis of the isotopic fingerprint of stable (C, N, O, H, S) and/or radiogenic (Sr) isotopes is one of the most common methods for the authentication of seafood products, especially when they originate from different countries and/or continents. Herein, we showed that the use of a single isotopic parameter (e.g., ^87^Sr/^86^Sr) or a combination of two isotopic parameters (e.g., δ^13^C vs. δ^15^N) is enough to trace the provenance of samples at the regional scale. However, a valid protocol for local scale traceability is missing, which poses a risk for human health, as in complex environmental settings single areas can be affected by different magnitudes of anthropic inputs (e.g., industrial and/or agricultural waste). Therefore, for the first time, we tested the reliability of this approach in distinguishing manila clams (*Ruditapes philippinarum*) collected in three lagoons in the same locality along the northern Adriatic coast in Italy (Sacca di Goro, Sacca di Scardovari, Comacchio Lagoon). Despite these lagoons sharing the same climatic conditions and water (Po River and Adriatic Sea) and feeding (marine and continental organic compounds) systems, the isotopic ratios of C, N, and S of the tissues and C, O, and Sr of the shells of manila clams highlighted the different marine and continental contributions in terms of both water and nutrient supplies. According to the stable isotope fingerprint of tissues and shells, samples collected at Sacca di Scardovari and Sacca di Goro (2018), including those bought at the local supermarket, record marine contributions, as these lagoons are well connected to the sea, whereas those from the Comacchio Lagoon mainly record the continental contribution. The S, C, and O isotopic composition of Sacca di Goro (2015) reflects the continental contribution related to a flood that occurred in spring of that year. For all the lagoons, the Sr isotopic ratios were close to the typical marine value, due to the abundance of this element in the sea as compared to rivers. However, the application of a linear discriminant analysis (LDA) to the stable isotope compositions of the manila clam tissues and shells allowed for the identification of the organisms’ lagoons of provenance. Therefore, this work demonstrates that, if combined, the differences in isotopic ratios of C, N, O, and S of manila clam tissues and shells collected close in space and time are relevant and make it possible to identify the exact lagoon of provenance of manila clams. This sheds light on the need of: (i) an exhaustive archive to emphasize isotopic differences and analogies between the food commodities collected in different locations and periods, and (ii) the promotion of a standardized multi-isotope analytical method for the authentication of seafood to combat fraud at both the regional and local scale. Aside from the study of traceability, this work provides interpretations for the variability of each isotopic parameter, which can also be useful for other geochemical studies in terms of selecting the correct isotopic parameter(s) for further investigations of specific features of lagoons (e.g., types of nutrient supply and water circulation).

## Figures and Tables

**Table 1:** Elemental and isotopic compositions of carbon (C), nitrogen (N), sulfur (S), oxygen (O), and strontium isotope composition (^87^Sr/^86^Sr) in tissues and shells of manila clam samples collected on-site from northern Adriatic lagoons and bought at the local supermarket. Analytical uncertainties for ^87^Sr/^86^Sr analyses are also reported.

Lagoon	Sample	Tissues							Shell		
		C (wt%)	N (wt%)	C/N	S (wt%)	δ^13^C (‰)	δ^15^N (‰)	δ^34^S (‰)	δ^13^C (‰)	δ^18^O (‰)	^87^Sr/^86^Sr ± 2σ
Sacca di Goro (2015)											
	G10	34.10	7.33	4.65	0.87	−24.0	+7.3	+17.5	−5.0	−3.6	0.709178 ± 0.000007
	G1	35.80	7.96	4.50	1.20	−22.9	+8.1	+13.6	−5.1	−3.5	
	G2	38.30	7.92	4.84	0.99	−23.6	+8.9	+16.7	−5.2	−4.1	
	G3	33.40	7.54	4.43	0.74	−23.8	+10.0	+13.5	−5.0	−4.0	
	G4	36.80	8.18	4.50	1.05	−23.8	+9.6	+16.6	−5.8	−3.9	0.709176 ± 0.000007
	G5	39.40	8.21	4.80	0.91	−24.7	+10.4	+17.2	−5.6	−3.4	
	G6	40.30	8.77	4.60	1.15	−24.1	+11.0	+17.3			
	G7	32.20	7.45	4.32	0.98	−22.7	+9.7	+15.6	−5.1	−3.5	
	G8	34.20	7.86	4.35	0.93	−23.7	+10.2	+16.7	−4.8	−3.7	0.709163 ± 0.000007
	G9	36.70	7.67	4.78	0.77	−23.7	+11.4	+17.1	−4.8	−3.2	
Sacca di Goro (2018)											
	GA	35.80	8.87	4.04	1.44	−21.5	+10.5	+19.2	−4.4	−3.4	0.709183 ± 0.000007
	GB	34.67	8.90	3.89	1.45	−21.4	+10.1	+19.4	−3.7	−3.4	
	GC	39.80	9.62	4.14	1.32	−21.7	+10.4	+19.9	−3.6	−3.7	
	GD	36.03	9.07	3.97	1.31	−21.3	+10.1	+19.7	−3.5	−3.9	0.709168 ± 0.000007
Supermarket (from Sacca di Goro; 2018)											
	TA	37.06	9.89	3.75	1.58	−20.4	+9.4	+19.5	−3.3	−2.5	
	TB	32.79	8.45	3.88	1.85	−20.2	+9.3	+20.5	−4.0	−2.9	0.709179 ± 0.000007
	TC	35.20	9.70	3.80	1.66	−20.8	+11.1	+20.2	−3.9	−2.6	
	TD	35.22	9.20	3.83	1.79	−20.2	+9.4	+19.8	−4.3	−3.9	
Sacca di Scardovari (2016)											
	FF1	36.90	7.63	4.84		−20.4	+7.2		−3.4	−3.0	
	FF2	37.24	7.68	4.85		−21.9	+7.9		−3.5	−2.9	0.709168 ± 0.000008
	FF4	31.35	6.90	4.54	1.19	−20.4	+8.8	+18.3	−2.7	−2.9	0.709176 ± 0.000006
	FF5	30.94	6.75	4.58	1.32	−20.4	+8.2	+19.0	−2.5	−1.5	0.709184 ± 0.000006
Laguna di Comacchio (2017)											
	LC1	38.52	8.87	4.34	1.36	−23.6	+14.7	+17.0	−5.1	−2.8	0.709177 ± 0.000007
	LC2	38.65	7.95	4.86	1.07	−24.7	+15.2	+14.2	−4.7	−3.3	0.709173 ± 0.000007
	LC3	36.83	8.72	4.22	1.45	−23.0	+14.2	+17.8	−2.4	−3.4	0.709177 ± 0.000005

## Data Availability

The data presented in this study are available on request from the corresponding author.

## References

[1] Kelly S., Heaton K., Hoogewerff J. (2005). Tracing the geographical origin of food: The application of multi-element and multi-isotope analysis. Trends Food Sci. Technol..

[2] Fox M., Mitchell M., Dean M., Elliot C., Campbell K. (2018). The seafood supply chain from a fraudulent perspective. Food Secur..

[3] Oehlenschläger J. (2012). Seafood: Nutritional benefits and risk aspects. Int. J. Vitam. Nutr. Res..

[4] Camin F., Bontempo L., Heinrich K., Horacek M., Kelly S.D., Schlicht C., Thomas F., Monahan F.J., Hoogewerff J., Rossmann A. (2007). Multi-element (H, C, N, S) stable isotope characteristics of lamb meat from different European regions. Anal. Bioanal. Chem..

[5] Schellenberg A., Chmielus S., Schlicht C., Camin F., Perini M., Bontempo L., Heinrich K., Kelly S.D., Rossmann A., Thomas F. (2010). Multielement stable isotope ratios (H, C, N, S) of honey from different European regions. Food Chem..

[6] Marchionni S., Braschi E., Tommasini S., Bollati A., Cifelli F., Mulinacci N., Mattei M., Conticelli S. (2013). High-precision ^87^Sr/^86^Sr analyses in wines and their use as a geological fingerprint for tracing geographic provenance. J. Agric. Food Chem..

[7] Braschi E., Marchionni S., Priori S., Casalini M., Tommasini S., Natarelli L., Buccianti A., Bucelli P., Costantini E.A.C., Conticelli S. (2018). From vine to wine: Data on 87Sr/86Sr from rocks and soils as a geologic and pedologic characterization of vineyards. Sci. Total Environ..

[8] Durante C., Baschieri C., Bertacchini L., Cocchi M., Sighinolfi S., Silvestri M., Marchetti A. (2013). Geographical traceability based on ^87^Sr/^86^Sr indicator: A first approach for PDO Lambrusco wines from Modena. Food Chem..

[9] Mimmo T., Camin F., Bontempo L., Capici C., Tagliavini M., Cesco S., Scampicchio M. (2015). Traceability of different apple varieties by multivariate analysis of isotope ratio mass spectrometry data. Rapid Commun. Mass Spectrom..

[10] Tescione I., Casalini M., Marchionni S., Braschi E., Mattei M., Conticelli S. (2020). Conservation of ^87^Sr/^86^Sr during wine-making of white wines: A geochemical fingerprint of geographical provenance and quality production. Front. Environ. Sci..

[11] Won E.-J., Kim S.H., Go Y.-S., Kumar K.S., Kim M.-S., Yoon S.-H., Bayon G., Kim J.-H., Shin K.-H. (2021). A Multi-Elements Isotope Approach to Assess the Geographic Provenance of Manila Clams (*Ruditapes philippinarum*) via Recombining Appropriate Elements. Foods.

[12] Turolla E., Castaldelli G., Fano E.A., Tamburini E. (2020). Life Cycle Assessment (LCA) Proves that Manila Clam Farming (*Ruditapes Philippinarum*) is a fully sustainable aquaculture practice and a carbon sink. Sustainability.

[13] Bianchini G., Brombin V., Carlino P., Mistri E., Natali C., Salani G.M. (2021). Traceability and authentication of manila clams from North-Western adriatic lagoons using C and N stable isotope analysis. Molecules.

[14] Marchina C., Bianchini G., Natali C., Pennisi M., Colombani N., Tassinari R., Knoeller K. (2015). The Po river water from the Alps to the Adriatic Sea (Italy): New insights from geochemical and isotopic (δ^18^O-δD) data. Environ. Sci. Pollut. Res..

[15] Marchina C., Bianchini G., Knoeller K., Natali C., Pennisi M., Colombani N. (2016). Natural and anthropogenic variations in the Po river waters (northern Italy): Insights from a multi-isotope approach. Isot. Environ. Health Stud..

[16] Marchina C., Bianchini G., Natali C., Knoller K. (2016). Geochemical and isotopic analyses on the Po delta water: Insights to understand a complex riverine ecosystem. Rend. Lincei Sci. Fis. Nat..

[17] Yokoyama H., Ishihi Y. (2006). Variation in δ^13^C and δ^15^N among different tissues of three estuarine bivalves: Implications for dietary reconstructions. Plankton Benthos Res..

[18] Wierzbowski H. (2007). Effects of pre-treatments and organic matter on oxygen and carbon isotope analyses of skeletal and inorganic calcium carbonate. Int. J. Mass Spectrom..

[19] Kusaka S., Nakano T. (2014). Carbon and oxygen isotope ratios and their temperature dependence in carbonate and tooth enamel using GasBench II preparation device. Rapid Commun. Mass Spectrom..

[20] Natali C., Bianchini G. (2015). Thermally based isotopic speciation of carbon in complex matrices: A tool for environmental investigation. Environ. Sci. Pollut. Res..

[21] Beccaluva L., Bianchini G., Natali C., Siena F. (2017). The alkaline-carbonatite complex of Jacupiranga (Brazil): Magma genesis and mode of emplacement. Gondwana Res..

[22] Dutta K., Schuur E.A.G., Neff J.C., Zimov S.A. (2006). Potential carbon release from permafrost soils of Northeastern Siberia. Glob. Change Biol..

[23] Coplen T.B., Qi H. (2011). USGS42 and USGS43: Human-hair stable hydrogen and oxygen isotopic reference materials and analytical methods for forensic science and implications for published measurement results. Forensic Sci. Int..

[24] Halas S., Szaran J. (2001). Improved thermal decomposition of sulfates to SO2 and mass spectrometric determinations of δ^34^S of IAEA-SO-5, IAEA-SO-6 and NBS-127 sulfate standards. Rapid Commun. Mass Spectrom..

[25] Avanzinelli R., Boari E., Conticelli S., Francalanci L., Guarnieri L., Perini G., Petrone C.M., Tommasini S., Ulivi M. (2005). High precision Sr, Nd, and Pb isotopic analyses using the new generation thermal ionisation mass spectrometer ThermoFinnigan Triton-Ti^®^. Period. Mineral..

[26] Weis D., Kieffer B., Maerschalk C., Barling J., De Jong J., Williams G.A., Hanano D., Pretorius W., Mattielli N., Scoates J.S. (2006). High-precision isotopic characterization of USGS reference materials by TIMS and MC-ICP-MS. Geochem. Geophys. Geosyst..

[27] Thirlwall M.F. (1991). Long-term reproducibility of multicollector Sr and Nd isotope ratio analysis. Chem. Geol..

[28] R Core Team R: A Language and Environment for Statistical Computing. https://www.R--project.org/.

[29] Briant N., Savoye N., Chovelon T., David V., Rodriguez S., Charlier K., Sonke J.E., Chiffoleau J.F., Brach-Papa C., Knoery J. (2018). Carbon and nitrogen elemental and isotopic ratios of filter-feeding bivalves along the French coasts: An assessment of specific, geographic, seasonal and multi-decadal variations. Sci. Total Environ..

[30] Dias E., Chainho P., Barrocas-Dias C., Adão H. (2019). Food sources of the non-indigenous bivalve *Ruditapes philippinarum* (Adams and Reeve, 1850) and trophic niche overlap with native species. Aquat. Invasions.

[31] Wang Z.-L., Zhang J., Liu C.-Q. (2007). Strontium isotopic compositions of dissolved and suspended loads from the main channel of the Yangtze River. Chemosphere.

[32] Tulli F., Moreno-Rojas J.M., Messina C.M., Trocino A., Xiccato G., Muñoz-Redondo J.M., Santulli A., Tibaldi E. (2020). The use of stable isotope ratio analysis to trace European sea bass (D. Labrax) originating from different farming systems. Animals.

[33] Martino J.C., Mazumder D., Gadd P., Doubleday Z.A. (2022). Tracking the provenance of octopus using isotopic and multi-elemental analysis. Food Chem..

[34] DeNiro M.J., Epstein S. (1978). Influence of diet on the distribution of carbon isotopes in animals. Geochim. Cosmochim. Acta.

[35] DeNiro M.J., Epstein S. (1981). Influence of diet on the distribution of nitrogen isotopes in animals. Geochim. Cosmochim. Acta.

[36] Zhao L., Yan X., Yang F. (2013). Food sources of the Manila clam *Ruditapes philippinarum* in intertidal areas: Evidence from stable isotope analysis. Chin. J. Oceanol. Limnol..

[37] Komorita T., Kajihara R., Tsutsumi H., Shibanuma S., Yamada T., Montani S. (2014). Food sources for *Ruditapes philippinarum* in a coastal lagoon determined by mass balance and stable isotope approaches. PLoS ONE.

[38] Bongiorni L., Nasi F., Florentino F., Auriemma R., Rampazzo F., Nordström M.C., Berto D. (2018). Contribution of deltaic wetland food sources to coastal macrobenthic consumers (Po River Delta, north Adriatic Sea). Sci. Total Environ..

[39] Berto D., Rampazzo F., Noventa S., Cacciatore F., Gabellini M., Bernardi Aubry F., Girolimetto A., Boscolo Brusà R. (2013). Stable carbon and nitrogen isotope ratios as tools to evaluate the nature of particulate organic matter in the Venice lagoon. Estuar. Coast. Shelf Sci..

[40] Yokoyama H., Tamaki A., Harada K., Shimoda K., Koyama K., Ishihi Y. (2005). Variability of diet-tissue isotopic fractionation in estuarine macrobenthos. Mar. Ecol. Prog. Ser..

[41] Abbiati M., Mistri M., Bartoli M., Ceccherelli V.U., Colangelo M.A., Ferrari C.R., Giordani G., Munari C., Nizzoli D., Ponti M. (2010). Trade-off between conservation and exploitation of the transitional water ecosystems of the northern Adriatic Sea. Chem. Ecol..

[42] Mistri M., Fano E.A., Rossi G., Caselli K., Rossi R. (2000). Variability in macrobenthos communities in the Valli di Comacchio, Northern Italy, a hypereutrophized lagoonal eco system. Estuar. Coast. Shelf Sci..

[43] Martinelli G., Dadomo A., De Luca D.A., Mazzola M., Lasagna M., Pennisi M., Pilla G., Sacchi E., Saccon P. (2018). Nitrate sources, accumulation and reduction in groundwater from Northern Italy: Insights provided by a nitrate and boron isotopic database. Appl. Geochem..

[44] Watanabe S., Katayama S., Kodama M., Cho N., Nakata K., Fukuda M. (2009). Small-scale variation in feeding environments for the Manila clam *Ruditapes philippinarum* in a tidal flat in Tokyo Bay. Fish. Res..

[45] Suh Y.J., Shin K.-H. (2013). Size-related and seasonal diet of the manila clam (*Ruditapes philippinarum*), as determined using dual stable isotopes. Estuar. Coast. Shelf Sci..

[46] Peterson B.J., Fry B. (1987). Stable isotopes in ecosystem studies. Annu. Rev. Ecol. Syst..

[47] Hesslein R.H., Hallard K.A., Ramlal P. (1993). Replacement of sulfur, carbon, and nitrogen in tissue of growing broad whitefish (*Coregonus nasus*) in response to a change in diet traced by δ^34^S, δ^13^C, and δ^15^N. Can. J. Fish. Aquat. Sci..

[48] Pinzone M., Acquarone M., Huyghebaert L., Sturaro N., Michel L.N., Siebert U., Das K. (2017). Carbon, nitrogen and sulphur isotopic fractionation in captive juvenile hooded seal (*Cystophora cristata*): Application for diet analysis. Rapid Commun. Mass Spectrom..

[49] Connolly R.M., Guest M.A., Melville A.J., Oakes J.M. (2004). Sulfur stable isotopes separate producers in marine food-web analysis. Oecologia.

[50] Godbout L., Trudel M., Irvine J.R., Wood C.C., Grove M.J., Schmitt A.K., McKeegan K.D. (2010). Sulfur isotopes in otoliths allow discrimination of anadromous and non-anadromous ecotypes of sockeye salmon (*Oncorhynchus nerka*). Environ. Biol. Fishes.

[51] Nehlich O., Borić D., Stefanović S., Richards M.P. (2010). Sulphur isotope evidence for freshwater fish consumption: A case study from the Danube Gorges, SE Europe. J. Archaeol. Sci..

[52] Burke A., Present T.M., Paris G., Rae E.C.M., Sandilands B.H., Gaillardet J., Peucker-Ehrenbrink B., Fischer W.W., McClelland J.M., Spencer R.G.M. (2018). Sulfur isotopes in rivers: Insights into global weathering budgets, pyrite oxidation, and the modern sulfur cycle. Earth Planet. Sci. Lett..

[53] Mitchell M.J., Mayers B., Bailey S.W., Hornbeck J.W., Alewell C., Driscoll C.T., Likens G.E. (2001). Use of stable isotope ratios for evaluating sulfur sources and losses at the hubbard brook experimental forest. Water Air Soil Poll..

[54] Giani M., Berto D., Zangrando V., Castelli S., Sist P., Urbani R. (2005). Chemical characterization of different typologies of mucilaginous aggregates in the Northern Adriatic Sea. Sci. Total Environ..

[55] Borrell A., Gazo M., Aguilar A., Raga J.A., Degollada E., Gozalbes P., García-Vernet R. (2021). Niche partitioning amongst northwestern *Mediterranean cetaceans* using stable isotopes. Prog. Oceanogr..

[56] Salani G.M., Brombin V., Natali C., Bianchini G. (2021). Carbon, nitrogen, and sulphur isotope analysis of the Padanian Plain sediments: Backgrounds and provenance indication of the alluvial components. Appl. Geochem..

[57] Holmer M., Hasler-Sheetal H. (2014). Sulfide intrusion in seagrasses assessed by stable sulfur isotopes-a synthesis of current results. Front. Mar. Sci..

[58] Natali C., Bianchini G. (2017). Geochemical proxies of sediment provenance in alluvial plains with interfering fluvial systems: A study case from NE Italy. Catena.

[59] Coletta P., Pentecost A., Spiro B. (2001). Stable isotopes in charophyte incrustations: Relationships with climate and water chemistry. Palaeogeogr. Palaeoclimatol. Palaeoecol..

[60] Kaandorp R.J.G., Vonhof H.B., Del Busto C., Wesselingh F.P., Ganssen G.M., Marmól A.E., Romero Pittman L., van Hinte J.E. (2003). Seasonal stable isotope variations of the modern Amazonian freshwater bivalve *Anodontites trapesialis*. Palaeogeogr. Palaeoclimatol. Palaeoecol..

[61] Andrews J.E., Coletta P., Pentecost A., Riding R., Dennis S., Dennis P.F., Spiro B. (2004). Equilibrium and disequilibrium stable isotope effects in modern charophyte calcites: Implications for palaeoenvironmental studies. Palaeogeogr. Palaeoclimatol. Palaeoecol..

[62] Shanahan T.M., Pigati J., Dettman D.L., Quade J. (2005). Isotopic variability in the aragonite shells of freshwater gastropods living in springs with nearly constant temperature and isotopic composition. Geochim. Cosmochim. Acta.

[63] Poulain C., Lorrain A., Mas R., Gillikin D.P., Dehairs F., Robert R., Paulet Y.-M. (2010). Experimental shift of diet and DIC stable carbon isotopes: Influence on shell  ^13^C values in the Manila clam *Ruditapes philippinarum*. Chem. Geol..

[64] Hoffman P.F., Lamothe K.G. (2019). Seawater-buffered diagenesis, destruction of carbon isotope excursions, and the composition of DIC in Neoproterozoic oceans. Proc. Natl. Acad. Sci. USA.

[65] Castorina F., Vaiani S.C. (2018). Riverine influence in Sr isotope ratio of mollusk shells and relationship with foraminiferal assemblages in a late Quaternary succession of the Po River Delta (northern Italy). Ital. J. Geosci..

[66] Marchina C., Natali C., Fahnestock F., Pennisi M., Bryce J., Bianchini G. (2018). Strontium isotopic composition of the Po river dissolved load: Insights into rock weathering in Northern Italy. Appl. Geochem..

[67] Lugli F., Cipriani A., Bruno L., Ronchetti F., Cavazzuti C., Benazzi S. (2022). A strontium isoscape of Italy for provenance studies. Chem. Geol..

[68] Allègre C.J., Louvat P., Gaillardet J., Meynadier L., Rad S., Capmas F. (2010). The fundamental role of island arc weathering in the oceanic Sr isotope budget. Earth Planet. Sci. Lett..

[69] Huang K.-F., You C.-F., Chung C.-H., Lin I.-T. (2011). Nonhomogeneous seawater Sr isotopic composition in the coastal oceans: A novel tool for tracing water masses and submarine groundwater discharge. Geochem. Geophys. Geosyst..

[70] Bernat M., Church T., Allègre C.J. (1972). Barium and strontium concentrations in Pacific and Mediterranean sea water profiles by direct isotope dilution mass spectrometry. Earth Planet. Sci. Lett..

[71] Mao Y., Lin F., Fang J., Fang J., Li J., Du M., Smaal A., Ferreira J., Grant J., Petersen J., Strand Ø. (2019). Bivalve Production in China. Goods and Services of Marine Bivalves.

[72] Wu L., Huh Y., Qin J., Du G., van Der Lee S. (2005). Chemical weathering in the Upper Huang He (Yellow River) draining the eastern Qinghai-Tibet Plateau. Geochim. Cosmochim. Acta.

[73] Wang J., Chapman D., Xu J., Wang Y., Gu B. (2018). Isotope niche dimension and trophic overlap between bigheaded carps and native filter-feeding fish in the lower Missouri River, USA. PLoS ONE.

[74] Wang X., Tang Z. (2020). The first large-scale bioavailable Sr isotope map of China and its implication for provenance studies. Earth Sci. Rev..

[75] Kuznetsov A.B., Semikhatov M.A., Gorokhova I.M. (2012). The Sr Isotope Composition of the World Ocean, Marginal and Inland Seas: Implications for the Sr Isotope Stratigraphy. Stratigr. Geol. Correl..

[76] Berrueta L.A., Alonso-Salces R.M., Héberger K. (2007). Supervised pattern recognition in food analysis. J. Chromatogr. A.

[77] Cuchet A., Anchisi A., Telouk P., Yao Y., Schiets F., Fourel F., Clément Y., Lantéri P., Carénini E., Jame P. (2021). Multi-element (^13^C, ^2^H and ^34^S) bulk and compound-specific stable isotope analysis for authentication of Allium species essential oils. Food Control.

